# Helmet wearing detection algorithm based on improved YOLOv5

**DOI:** 10.1038/s41598-024-58800-6

**Published:** 2024-04-16

**Authors:** Yiping Liu, Benchi Jiang, Huan He, Zhijun Chen, Zhenfa Xu

**Affiliations:** 1 School of Mechanical Engineering, Anhui Polytechnic University, Wuhu, 241000 People’s Republic of China; 2 School of Artificial Intelligence, Anhui Polytechnic University, Wuhu, 241000 People’s Republic of China; 3 Yangtze River Delta HIT Robot Technology Research Institute, Wuhu, 241000 People’s Republic of China; 4 AnHui Key Laboratory of Detection Technology and Energy Saving Devices, AnHui Polytechnic University, Wuhu, 241000 People’s Republic of China

**Keywords:** Deep learning, Target detection, YOLOv5, Network structure, Attention mechanism, Electrical and electronic engineering, Computer science

## Abstract

In industrial production, workers need to wear safety helmets at all times. However, due to different lighting, viewing angles, and the tendency of people to block each other, the precision of target detection is not high enough. Aiming at this problem, a real-time detection of helmets was achieved by improving the YOLOv5 algorithm. This algorithm introduces the lightweight network structure FasterNet, which uses partial convolution as the main operator to reduce the amount of calculations and parameters of the network; the boundary regression loss function Wise-IoU loss function with a dynamic focusing mechanism replaces the original loss function in YOLOv5; finally, the CBAM attention mechanism is introduced to obtain global context information and improve the detection ability of small targets. The experimental results show that the parameters of the improved YOLOv5 model are reduced by 12.68%, the computational amount is reduced by 10.8%, the mAP is increased from 88.3 to 92.3%, and the inference time is reduced by 81.5%, which is better than the performance of the original model and can detect helmet wearing effectively and in real time.

## Introduction

As a common and practical personal protective equipment, hard hats can effectively reduce or avoid accidental injuries to workers' heads. However, in most construction scenarios, hard hat wearing inspection is used manually. This method is not only time-consuming and labor-intensive but also it is easy to produce errors. In order to solve the safety management problem at the construction site, intelligent management of the site is implemented, so the detection and research on the wearing of safety helmets has important practical significance. As a commonly used personal protective equipment, safety hats can effectively mitigate or avoid accidental injuries to workers' heads, however, in most construction scenarios in our country, artificial use of safety hat wearing testing is not only time-consuming but also vulnerable to errors.

In recent years, the rapid development of computer vision technology, introduced such as RCNN series^1^, SSD series^2^, and You Only Look Once(YOLO) series^3^ and some high-performance target detection algorithms^4^. The RCNN series algorithm is the mainstream two-layer detection algorithm, the first phase of which mainly uses the Region Proposal to generate post-selection zones and extract characteristic vectors from them^5^; The second phase utilizes a convolution neural network to predict the location and category of the target, thereby achieving accurate detection and positioning of the goal. SSD series and YOLO series algorithms are the most popular single-phase detection algorithms today, they do not need to generate candidate areas, but instead enter the image from the input end and then output the target's position and category at the output end, this end-to-end technology greatly improves the detection performance of the algorithm^6^.In recent years, many scholars have tried to apply computer vision technology to judge the detection of wearing a safety hat^7^. In 2018, Fang etc.^8^ took the lead in applying the Faster RCNN algorithm to safety hat wear detection, although the precision of identification has improved, but still fails to meet the demand for real-time. In 2022, Van etc.^9^ proposed using YOLOv5 and YOLOR to detect hard hats at construction sites, but the precision of this algorithm was low. In 2023, Athidhi etc.^10^ proposed a safety helmet detection model based on YOLOv7, which has a good precision rate, but missed detection often occur when detecting small targets. Zhang etc.^11^ proposed safety helmet wear detection based on particle swarm optimization YOLOv7. This method has high recognition precision, but has high computational cost and large number of iterations.

In summary, there are still many problems in the research on safety helmet detection at home and abroad. At present, the research center is biased towards improving the detection precision, ignoring the limitations under actual construction conditions. Therefore, the deployment of lightweight target detection networks on embedded equipment has become a new point of special interest. In order for the algorithm to be deployed to the industrial site, to solve the limitations of storage space and power consumption, it is necessary to reduce the number of parameters and calculations of the target detection model. Experimentally proved that the reduction of the number is often accompanied by a large decrease in the model precision, so that the network can not be arbitrarily modified by seeking a reduction in model parameters, ignoring the requirements of precision and precision.

In this paper, two types of targets, construction workers wearing helmets and construction workers not wearing helmets, are taken as the detection task, and the helmet detection dataset is constructed. YOLOv5 is selected as the main body of the algorithm, firstly, the lighter weight network structure FasterNet^12^ is used to reduce the model computation and the number of parameters, in order to improve the convergence ability of the model the boundary regression loss function Wise-IoU loss function with dynamic focusing mechanism is used to replace the original loss function of YOLOv5, and the CBAM attention mechanism is introduced in the head to enhance the ability of obtaining the global context and improve the model detection precision. The experimental results show that the improved YOLOv5 algorithm has significantly higher mean average precision, better image processing speed than mainstream algorithms, and significantly lower number of parameters and computation, which can be effectively deployed on embedded devices to meet the requirements of detection in construction scenarios.

## Related work

**Figure 1:**
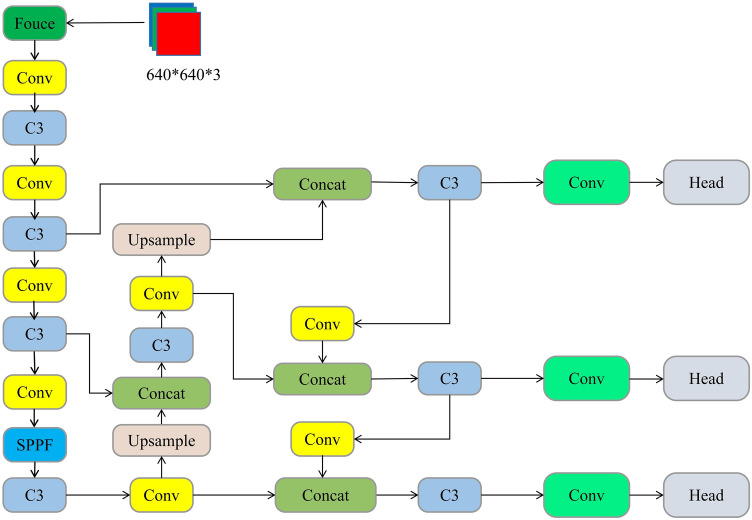
YOLOv5s structural chart.

YOLO is one of the most widely used target detection models, and the YOLOv5 detection algorithm is a lightweight model released by Ultralytics in 2020. YOLOv5 has four structures, namely Yolov5s, YOLOw5m, Yolow5l, and YOLOV5x, which control the four structure through the two parameters depth_multiple and width_multiple. YOLOv5s, as shown in Fig. 1, includes the input edge, the Backbone, the Neck, and the Prediction.

### Backbone

Backbone in YOLOv5 uses CSPDarknet53, which is an improved version based on Darknet53. Its main feature is the introduction of the Cross Stage Partial Network (CSP) module to reduce the number of parameters and calculations and improve model efficiency and precision. The core idea of the CSP module is to divide the feature map into two parts. One part is directly added to the other part after passing through the convolution layer, and then output through the convolution layer.

Based on the CSPNet^13^ design approach, YOLOv5s has designed two different CSP structures in the main core network and the multi-scale character integration network. Backbone uses CSP structure to first divide the original input into two branches, performing volume operations and N residual block operations, respectively, and finally stack the two branch operations to protect precision while reducing the amount of calculations.

### Neck (multi-scale feature integration network)

The YOLOv5 network-type mid-neck network (Neck) part uses the SPP + PANet^14^ design approach. The main responsibility for character enhancement is to enhance the processing of features extracted from the main network to increase the precision of the forecast. SPP is the abbreviation for space pyramid pooling, which first reduces the input channel to half through a standard pooling module, and then performs a maximum pooling of the volume core size of 5, 9, 13 respectively, and connects the results of the three maximum pooling with the data of non-pooling operations. The SPP module increases the connectivity of core features more effectively and significantly separates the most important contextual information. PANet consists of FPN + PAN, a pyramidal feature extraction network that can extract characteristics with different scales from different levels of characteristics, thereby increasing the precision of target detection.

### Head

YOLOv5 losses include Classification Loss, Localization Loss and Confidence Loss. Classification losses are used to measure whether the categories of objects in the predictive box are correct, and YOLOv5 uses the CrossEntropy Loss^15^ function to calculate the difference between the probability distribution of the object category in the Predictive Box and the real category. Position loss is used to measure the position precision of the forecast box, and YOLOv5 uses the Square Error Loss function to calculate the difference between the position coordinates of forecasts and the real location coordinates. Confidence loss is used to measure whether an object is contained in a predictive box, and YOLOv5 uses the BinaryCross Entropy Loss function to calculate the difference between the probability distribution of an object and the real label. Among them, classified losses and credibility losses are weighed less, while positioned losses have been weighed more to ensure that the position of the forecast box is more accurate.

## Improvements to the Yolov5s algorithm

**Figure 2:**
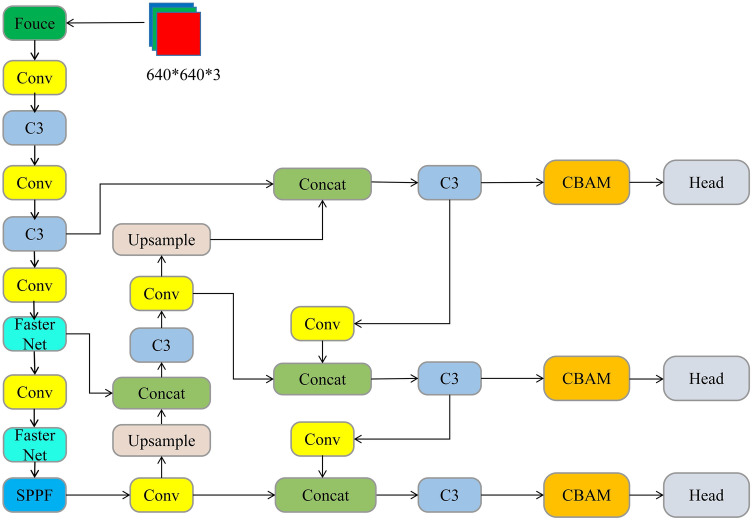
Improved YOLOv5s model.

The YOLOv5s has been improved to meet the requirements of real-time and precision for embedded device deployment conditions and on-site operations, without losing its original precision: (1) Replace the main core network with the lightweight network FasterNet for the problem of the large number of parameters and computing volume of the YOLOv5 model; (2) use the attention-based loss function Wise-IoU based on border regression (BBR); (3) introduce a CBAM attention mechanism between the Neck and Head, respectively, to enhance network performance. The improved YOLOv5s model is shown in Fig. 2.

### Improvements to the main actors network

**Figure 3:**
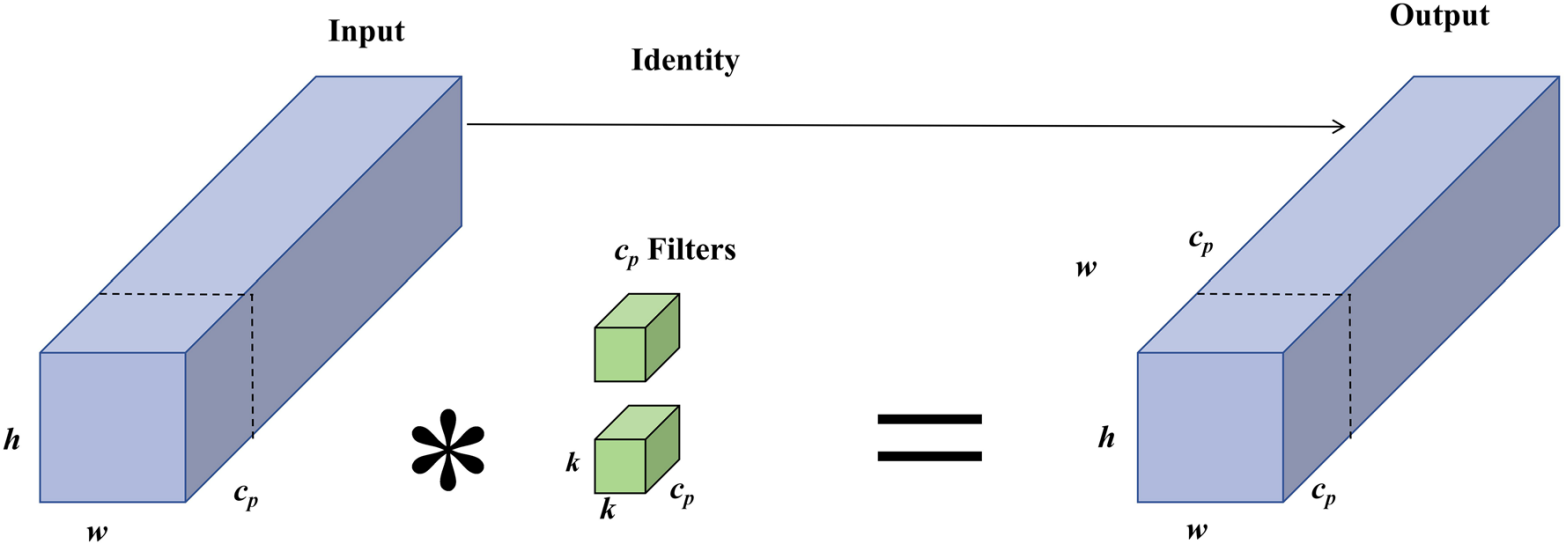
Partial convolution structural chart.

The YOLOv5 core network uses CSPDarknet53 to extract characteristics, containing a large number of deep-voltage modules and computing volumes. Lightweight network models reduce model parameters by designing more efficient network computing methods. The FasterNet network is a lightweighted neural network that achieves fast computation by reducing FLOPS. The basic component of FasterNet is Partial Convolution (PConv), which is structured as shown in Fig. 3.

**Figure 4:**
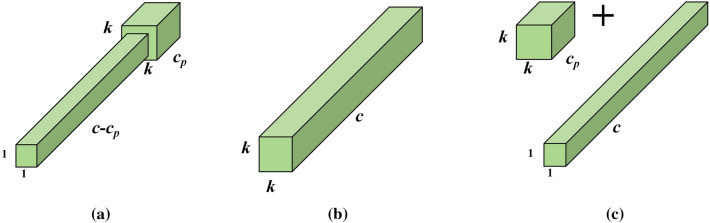
(**a**) T-shaped Conv (**b**) Regular Conv (**c**) PConv + PWConv.

It only applies the conventional Conv to a portion of the input channel to extract spatial characteristics and keeps the rest of the channel unchanged, with PConv's FLOPs (floating point counting) being only $h \times w \times k^{2} \times c_{p}^{2}$. To make full and effective use of information from all channels, PWConv is further added to the partial volume (PConv). The effective sensing field on their input attribute chart looks like a T-shaped Conv, as shown in Fig. 4a, compared with the Regular Conv as shown on Fig. 4b, which is more focused in the center position.

In order to justify this T-shaped receptive field, the importance of each position is first evaluated by calculating the position Frobenius norm in^19^. It is assumed that a position tends to be more important if it has a larger Frobenius norm than any other position. For Conv filter ${\text{F}} \in {\text{R}}^{{k^{2} \times c}}$, the Frobenius parameter for position i is calculated as in Eq. (1).

1 $$\left\| {F_{i} } \right\| = \sqrt {\sum\nolimits_{j = 1}^{c} { \, \left| {f_{ij} } \right|^{2} } } \quad i = 1,2,3....,k^{2}$$

**Figure 5:**
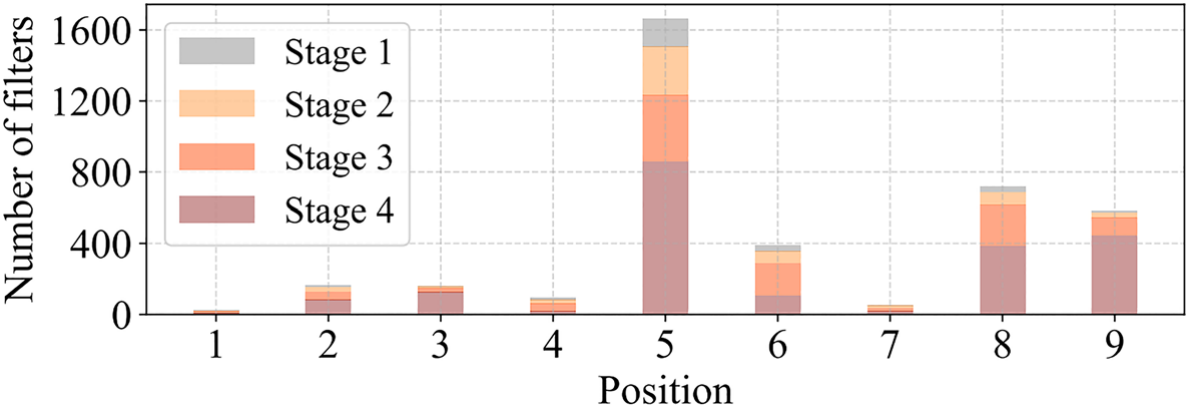
Distributed rectangular distribution of the distinctive position of conventional Conv 3 × 3 filters in pre-trained ResNet18. The diagram contains four bar diagrams corresponding to different stages of the network. In all stages, the central position (position 5) is the most prominent position.

Each filter was then jointly inspected in the pre-trained ResNet18 to identify their prominent location and draw a diagram of the prominent position, as shown in Fig. 5.

The result indicates that the center position is the most commonly observed position in the filter. In other words, the central position is more important than the surrounding position, which is consistent with the T-shape calculation that is concentrated in the center position. While T-shape Conv can be directly used for efficient computing, decomposing T- shape Conve to PConv and PWConv, as shown in Fig. 4c, can decompose redundancy between filters, further saving FLOP. For the same input ${\text{I}} \in {\text{R}}^{{{\text{c}} \times h \times w}}$ and $O \in R^{c \times h \times w}$, a T-shaped Conv's FLOPs can be counted as Eq. (2), higher than the PConv and PWConv's FLOPs that are Eq. (3).

2 $$h \times w \times \left( {k^{2} \times c_{p} \times c + c \times (c{ - }c_{p} )} \right)$$

3 $${\text{h}} \times w \times \left( {k^{2} \times c_{p}^{2} + c \times c_{p} } \right)$$

**Figure 6:**
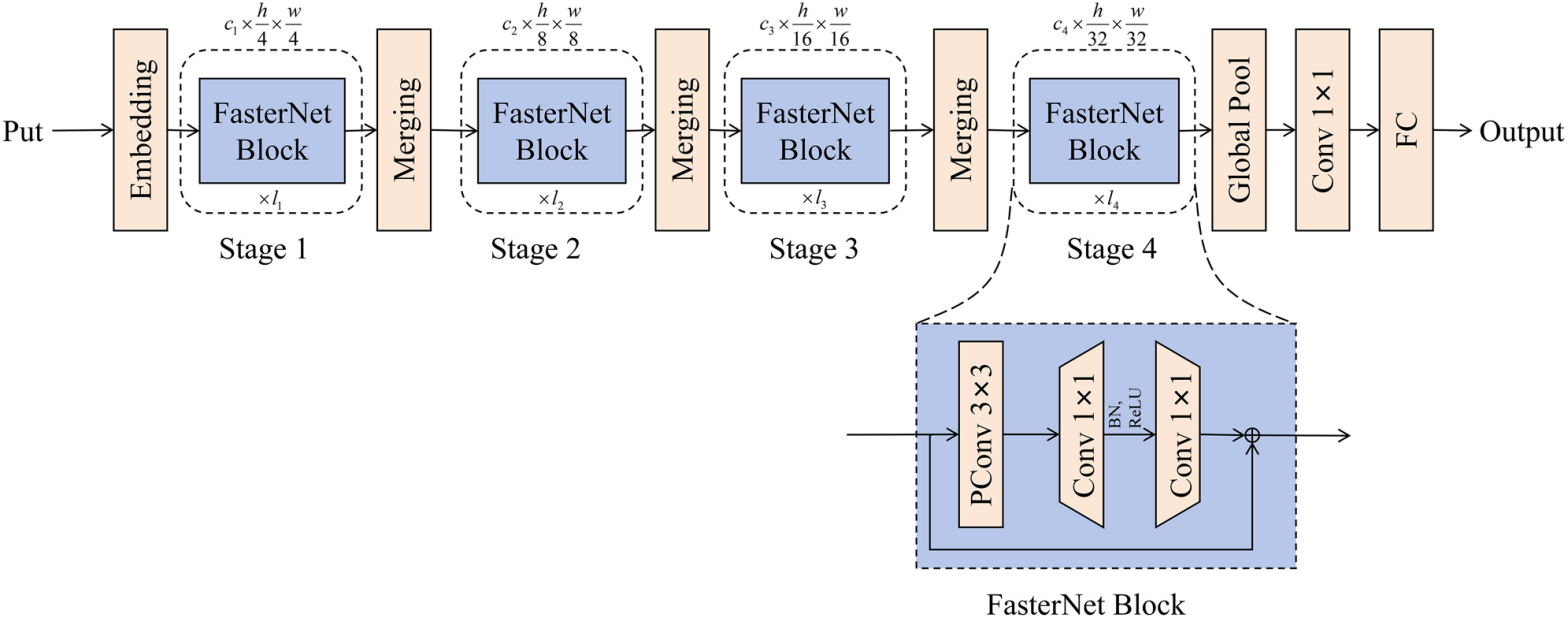
FasterNet has four layer stages, each with a stack of fast grid blocks with an embedded or merged layer in front of it. The last three layers are used for character classification. In each FasterNet block, there are two PWConv layers behind a PConv layer. We only place the unification layer and the activation layer behind the middle layer to maintain characteristic diversity and a lower latency.

Among them ${\text{c}} > {\text{c}}_{{\text{p}}}$, ${\text{c}}{ - }c_{p} > c_{p}$. In addition, conventional Conv can be easily used for two-step implementation. The overall structure of the FasterNet network is shown in Fig. 6. It has four layered stages, with each stage preceded by an embedded layer (conventional Conv4 × 4, step width is 4) or a joint layer, conventional Conv2 × 2, stepwidth is 2) for sampling and expansion of channels under space. Each stage has a stack of FasterNet blocks, and more computations are assigned accordingly for the last two stages. Each FasterNet block has a PConv layer followed by two PWConv (or Conv1 × 1) layers.

### Replacement of the loss function

When detecting a target in the image, the algorithm will generate multiple predictive boxes because more than one target is present in the field of vision, which requires the removal of excess predictions using a non-extremely suppressive method^16^. The original YOLOv5 uses the GIoU_Loss function as a loss function^17^, the principle of which is found in Eq. (4).

4 $$R_{GIoU} = R_{IoU} { - }{{\left| {C{ - }\left( {A \cup B} \right)} \right|} \mathord{\left/ {\vphantom {{\left| {C{ - }\left( {A \cup B} \right)} \right|} {\left| C \right|}}} \right. \kern-0pt} {\left| C \right|}}$$

GIoU adds the intersecting scale trade-off method, which effectively solves the problem of non-overlapping boundaries. However, when the prediction box coincides with the target or the width and height are aligned, the GIoU loss function will degenerate during regression, so that the relative position of the target cannot be accurately predicted, resulting in an increase in the number of iterations and a slow detection speed^18^. This paper introduces a bounding box regression loss with a dynamic focusing mechanism proposed in^19^.

Since the training data inevitably contains low-quality examples, geometric factors such as distance and aspect ratio will aggravate the penalty of low-quality examples, thereby reducing the generalization performance of the model. A good loss function should weaken the penalty of geometric factors when the anchor box and the target box coincide well, and less intervention in training will lead to better generalization of the model. On this basis, the distance attention is constructed, and the WIoUv1 with a two-layer attention mechanism is obtained, as shown in the Eqs. (5) and (6).

5 $${\mathcal{L}}_{WIoUv1} ={\mathcal{R}}_{WIoU}{\mathcal{L}}_{IoU}$$

6 $${\mathcal{R}}_{WIoU} = {\text{exp}}\left( {\frac{{(x - xgt)^{2} + (y - ygt)^{2} }}{{(Wg^{2} + Hg^{2} )^{*} }}} \right)$$

**Figure 7:**
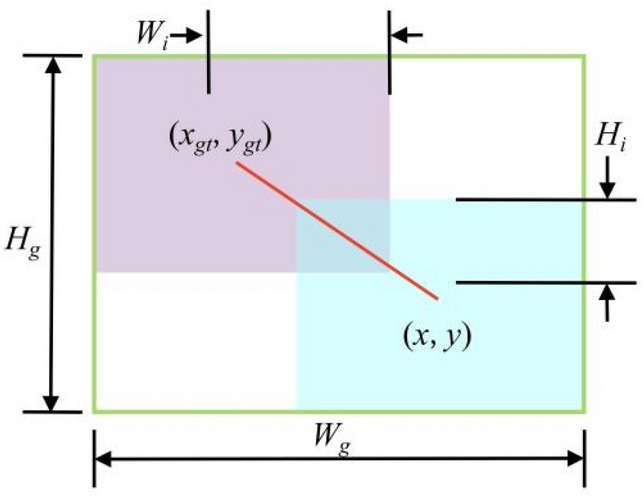
Minimum closed box and its center distance.

In the equation, *x* and *y* represent the center point coordinates of the predicted box; *x*_*gt*_ and *y*_*gt*_ represent the center point coordinates of the real box; *W*_*g*_ and* H*_*g*_ represent the width and height of the minimum closed box respectively. When ${\mathcal{R}}_{{WIoU}} \in \left[ {1,e} \right)$ will enlarge the ordinary quality prior box, when ${\mathcal{L}}_{{IoU}} \in \left[ {1,e} \right]$ will shrink the high quality prior box, when the prior box coincides with the target box, focus on the distance between its center points, as shown in Fig. 7.

In order to prevent the ${\mathcal{R}}_ WIoU$ gradient from disappearing, *W*_*g*_ and *H*_*g*_ are separated from the calculation, that is, the values of the variables *W*_*g*_ and *H*_*g*_ are fixed and converted into constants so that they do not participate in back propagation and prevent excessive obstruction of convergence (in the equation * represents this operation) .

### Introduction of attention mechanisms

**Figure 8:**
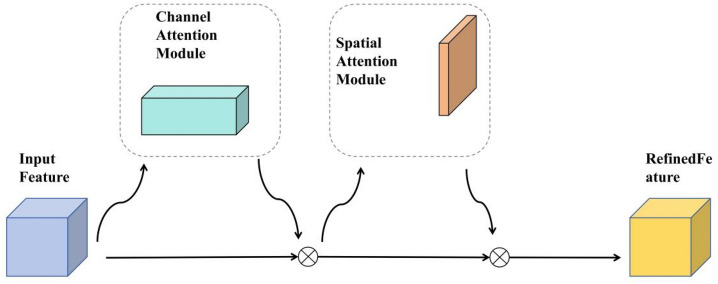
CBAM (convolutional block attention module) module structure.

The original YOLOv5s network often loses information about the safety hats of the small target when it comes to deep clustering. Thus, through the introduction of the CBAM^20^ attention mechanism, the linkage of location information in images with safety hat features has been further strengthened and the characteristic expression of important information in the network has been enriched. Its network structure is as shown in Fig. 8. The CBAM attention mechanism combines the Channel Attention Module and the Spatial Attentions Module to process the input character layer of the channel attention module and of the spatial attentions module respectively.

**Figure 9:**
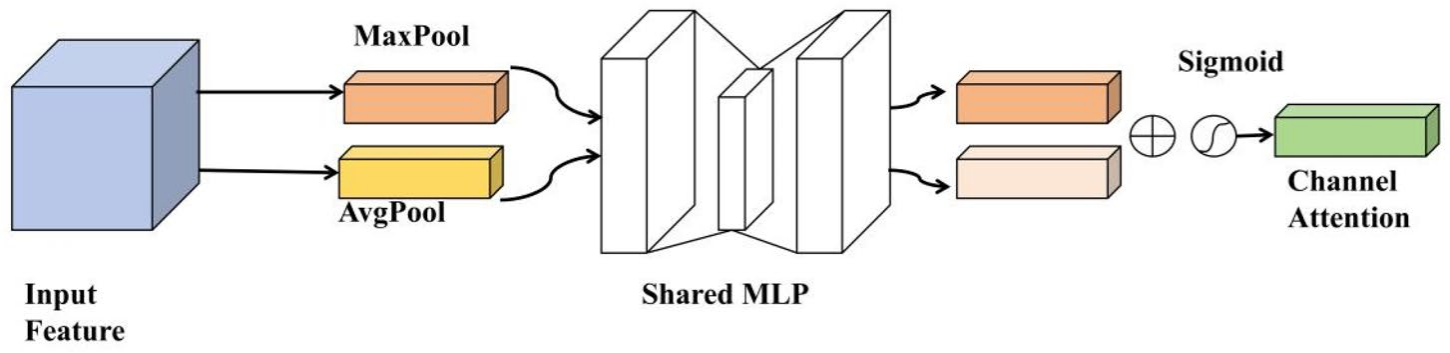
Channels attention module structure.

The channel attention module, as shown in Fig. 9, first uses the spatial information of the Average Pooling and Max Pooling operating polymer characteristics to generate the average poolization characteristics $F_{avg}^{C}$ and $F_{max}^{c}$. The two spatial descriptors are then forwarded to a shared network to generate Channel Attention Map $M_{c} \in R^{C \times 1 \times 1}$. A shared network consists of a multi-sensor (MLP) containing a hidden layer, and in order to reduce the call of the parameter, the hidden activation size is set to $R^{C/r \times 1 \times 1}$, where r is the reduction rate. The calculation process is as shown in Eq. (7), where b represents the Sigmoid function, $W_{0} \in R^{C \times C/r}$, $W_{1} \in R^{C \times C/r}$, and the weight of MLP is shared by W0 and W1 and $W_{0}$ is activated by the ReLU function before.

7 $$\begin{aligned} M_{C} (F) & = \sigma (MLP(AvgPool(F)) + MLP(MaxPool(F))) \\ & = \sigma (W_{1} (W_{0} F_{avg}^{c} )) + W_{1} (W_{0} (F_{max}^{c} ))) \\ \end{aligned}$$

**Figure 10:**
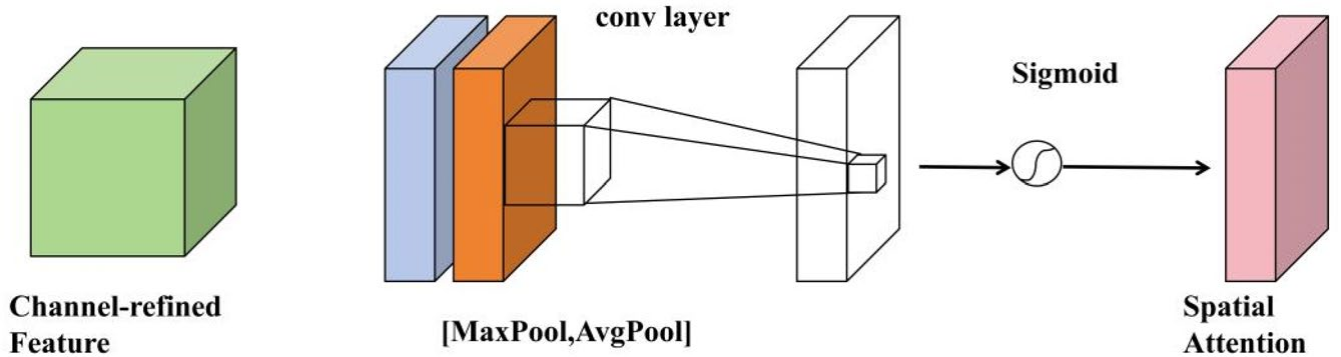
Spatial attention module structure.

Unlike channel attention modules, the space attention module focuses on which part of the input information is more important, and its structure is as shown in Fig. 10. First, through two pooling operations, the channel information of a characteristic chart is aggregated and two mappings are generated: $F_{avg}^{s} \in {\mathbf{R}}^{1 \times H \times W}$ and $F_{\max }^{s} {\mathbf{R}}^{1 \times H \times W}$, which represent the average pooling characteristics of the channels and the maximum polarization characteristics respectively. Finally, standardization is done with the Sigmoid function. The specific calculation equations is as shown in the Eq. (8), in which b represents the Sigmoid function, and ${\text{f}}^{7 \times 7}$ represents a compact nucleus with a size of 7 × 7.

8 $$\begin{aligned} M_{S} (F) & = \sigma (f^{7 \times 7} ([AvgPool(F);MaxPool(F)]) \\ & = \sigma (f^{7 \times 7} ([F_{avg}^{s} ;F_{max}^{s} ])) \\ \end{aligned}$$

## Experiments and analysis

### Data sets and experimental settings

The quality of the data set is crucial for the results of deep learning. Currently, the only open-source data set available is the Safety Helmet Wearing Dataset (SHWD). However, this data set lacks small objects in complex environments, and the images depicting the absence of safety helmets mainly come from non-construction site scenarios. Therefore, this data set is not a standard construction site data set and does not meet the real-time monitoring requirements in actual production environments.

This experiment uses a fused helmet data set, which consists of the SHWD data set, photographs of workers' operations taken in the field, and images crawled from the web, and it is each image scene in the data set that is a construction site scene. Use labelimg to label the image and save it in YOLO format. The data set has a total of 6892 pictures, the training set has 6202 pictures, and the test set has 690 pictures. The training set and the test set Strictly independent.

The experimental platform hardware environment in this article is NVIDIA RTX 3060 12 GB GPU, Intel Core i5-12400F CPU; operating system and software environment: Windows10, CUDA v12.0, cuDNN v8.8.0, python v3.8.16, pytorch v1.7.0. The size of the images sent to the web training is 640 × 640, batch size is set to 16, learning rate is 0.01, training is 100 epochs.

### Indicators of evaluation

In order to measure the performance of the algorithm, the detection precision, recall rate, average precision $mAP$ is used as the criterion for measuring the performance^21^, $mAP^{0.5:0.95}$ represents the total average value of $mAP$ when the threshold is between 0.5 and 0.95, the calculation can be calculated using the following equations.

9 $${\text{Precision}} = \frac{TP}{{TP + FP}}$$

10 $${\text{Re}} call = \frac{TP}{{TP + FN}}$$

11 $$mAP = \frac{1}{C}\sum\limits_{i = 1}^{c} {AP_{i} }$$

In the equations, the TP (True Positives) represents the positive sample has been correctly identified; the FN (False Negatives) represents the negative sample is erroneously recognized as negative; and the FP (False Positive) represents negative samples have been incorrectly recognized as positive. AP represents the average precision on a single category; C represents the number of categories.

### Ablation experiment

Ablation experiments are conducted on the same dataset for each improved module of YOLOv5 to test the effect of each improved module. The experiments are based on YOLOv5 with the introduction of FasterNet, the replacement of the loss function with the Wise-IoU loss function, and the introduction of the CBAM attention mechanism, and + indicates that an improvement of a module is added, as shown in Table 1.

**Table 1:** Results of ablation experiments.

Model	mAP@0.5 (%)	Parameters (M)	GFLOPS	Precision (%)	Inference time (ms)
YOLOv5s	88.3	7.02	15.8	83.0	27
YOLOv5s + FasterNet	88.9	6.10	14.1	84.4	4
YOLOv5s + Wise-IoU	88.4	7.02	15.8	83.7	5
YOLOv5s + CBAM	88.1	7.05	15.8	84.3	6
YOLOv5s + FasterNet + Wise-IoU	89.2	6.64	14.1	85.2	4
YOLOv5s + FasterNet + CBAM	89.8	6.72	14.1	87.5	5
YOLOv5s + Wise-IoU + CBAM	90.5	7.11	15.8	86.9	7
Ours	92.3	6.13	14.1	90.9	5

The original YOLOv5 model has a mean average precision of 88.3%, a parameter count of 7.02 M, a GFLOPS of 15.8, an precision rate of 83%, and an inference time of 27 ms. By introducing only the lightweight network structure FasterNet or replacing the original loss function with the Wise-IoU loss function, or combining the two, the parameter count, GFLOPS, and inference time of the model decrease significantly. YOLOv5s model adds CBAM attention mechanism, the mean average precision of the improved model decreases and the number of parameters rises, but the precision rate is significantly improved. From Table 1, we can see that the mean average precision of YOLOv5 + FasterNet + Wise-IoU combination reaches 89.2%, and the inference time is 4 ms, while the mean average precision of the algorithm proposed in this paper reaches 92.3%, and the inference time is 5 ms. adding CBAM attention mechanism improves the inference time slightly, but the mean average precision is significantly improved, and the comprehensive comparison of the algorithm proposed in this paper is the best. The algorithm proposed in this paper has the best effect. Comparing our algorithm to the original model, we achieved a 4% increase in mean average precision, a 12.68% reduction in parameter count, a 10.8% reduction in computation cost, and a 7.3% improvement in precision.

### Comparison experiments

In order to verify the effectiveness of the improved algorithm in this article, several popular target detection networks are used for comparative experiments: Faster RCNN, SSD, YOLOv4 Tiny, YOLOv7 and YOLOv8. The experimental process followed the principle of controlled variables, and the experimental software and hardware environment remained unchanged. The evaluation indicators use mean average precision (mAP), parameter volume, Giga Floating Point Operations Per Second (GFLOPS), precision, and inference time. The experimental results are shown in Table 2.

**Table 2:** Comparison of experimental results.

Model	mAP@0.5 (%)	Parameters (M)	GFLOPS	Precision (%)	Inference time (ms)
Faster RCNN	73.8	82.90	216.2	59.4	126
SSD	58.0	26.73	23.6	85.9	89
YOLOv4 Tiny	63.7	7.23	9.4	74.1	45
YOLOv7	90.2	3.67	103.2	87.8	8
YOLOv8	91.8	11.3	31.2	90.2	27
Ours	92.3	6.13	14.1	90.9	5

As shown in Table 2, the mean average precision of this paper's model on the constructed helmet dataset is 18.5%, 34.4%, 28.6%, 2.1%, and 0.5% higher than that of Faster RCNN, SSD, YOLOv4 Tiny, YOLOv7, and YOLOv8 algorithms, respectively, and it has a more excellent detection performance. The order of model computation size is YOLOv7 < Ours < YOLOv4 Tiny < YOLOv8 < SSD < Faster RCNN, and the order of inference time is Ours < YOLOv7 < YOLOv8 < YOLOv4 Tiny < SSD < Faster RCNN, and the experiment proves that this paper's algorithm meets the demand of real-time detection. Comprehensively, the improved algorithm has a lower number of parameters and computation volume compared with the latest YOLOv8, and the mean average precision and precision are still improved by 0.5% and 0.7% under the premise that the inference time is reduced by 22 ms. Compared with the traditional Faster RCNN and SSD models, the improved algorithm not only greatly reduces the number of parameters, computation volume, inference time, but also has a greater average precision and precision of the mean average. Precision and precision, etc. has a large improvement. Compared with YOLOv7, although the number of parameters is increased, the improved algorithm still reduces the inference time by 3 ms due to the greatly reduced computation amount, while the mean average precision and precision are improved by 2.1% and 3.1%, respectively. Compared with the lightweight network YOLOv4 Tiny there is a large improvement in mean average precision and precision. The comprehensive results in all aspects show that the improved YOLOv5 model in this paper has high mean average precision to satisfy the precision requirement, while the computational and parametric quantities are low, which is easy to be deployed in the edge devices.

### Test results

**Figure 11:**
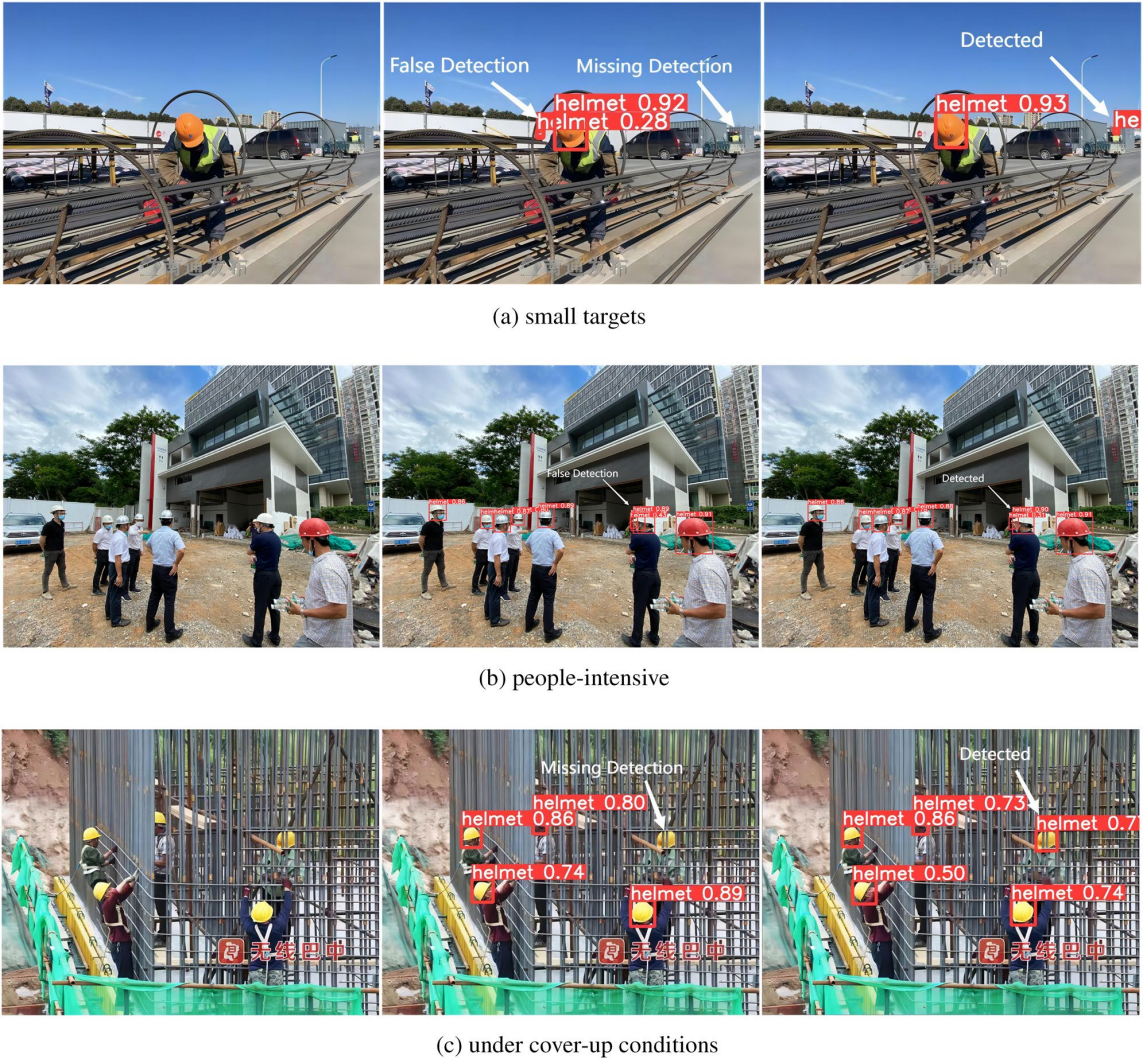
Test result comparison image.

In order to more intuitively validate the detection effect of the improved YOLOv5 model, this paper verifies the safety hat detection in different scenarios, including small targets (Fig. 11a) and people-intensive (Fig. 11b) and under cover-up conditions (Fig. 11c) and compares the results, comparing the results as shown in Fig. 11.

As shown in Fig. 11, the first picture in each group is the original picture, the second one is the original YOLOv5 detection result, and the third one is the detection result of the improved YOLOv5 in this paper. As the improved method in this paper introduces CBAM attention mechanism between neck and head and uses Wise-IoU, a boundary regression loss function with dynamic focusing mechanism, it helps the model to focus on important positions and information, and reduces missed and wrong detection in complex scenes. It is demonstrated that the improved YOLOv5 feature extraction capability has been enhanced and the detection performance in different environments has been improved.

## Conclusion

This paper uses the study of deep learning-based target detection as an input point to improve the YOLOv5 model by reducing the number of parameters and calculations of the model while meeting precision requirements, while improving the precision of recognition. First, the main core network is modified with FasterNet to reduce the number and calculation of the parameter model. In addition, the loss function of the original Yolov5 is replaced by the Wise-IoU loss function, which reduces the regression error of a model. Finally, the CBAM attention module is added between the Neck and the Head, which enhances the model's ability to acquire the semantic capacity in the context. Tested on the self-built data set, compared with the original YOLOv5s model, the number of parameters and computational volume of the improved model decreased, the mean average precision of the model increased by 4%, and the inference time was shortened to 5 ms. The test results proved that the improved algorithm in this paper is superior to the original YOLOv5s model, and it meets the requirements of helmet wearing detection in complex construction site scenarios. In subsequent research, the algorithm will be added with the capability of whether the helmet is worn correctly or not, and a face recognition system will be introduced to identify the target, so that construction workers who are not wearing helmets can be directly alerted.

## Data Availability

Data or code presented in this study are available request from the corresponding author.
